# Protein – a scoping review for Nordic Nutrition Recommendations 2023

**DOI:** 10.29219/fnr.v67.10261

**Published:** 2023-12-12

**Authors:** Ólöf Guðný Geirsdóttir, Anne-Maria Pajari

**Affiliations:** 1 Faculty of Food Science and Nutrition, School of Health Science, University of Iceland, Reykjavik, Iceland; 2 Department of Food and Nutrition, University of Helsinki, Helsinki, Finland

**Keywords:** dietary protein, amino acids, protein recommendations, essential amino acids, nitrogen balance, protein intake, protein turnover, protein bioavailability, protein quality

## Abstract

Proteins are needed for providing essential amino acids, nitrogen, and fuel for the body’s needs in all age groups. Proteins are especially required during active growth in pregnancy, lactation, childhood, and tissue growth in general. An adequate protein intake is needed in old adults to avoid premature muscle loss. According to the current dietary surveys, protein intake in the Nordic and Baltic countries varies from 15 to 19% of the total energy intake in adults. Comprehensive data regarding children and older adults are lacking. No good measure for protein status exists, and the estimation of physiological requirements is based on N-balance studies having some weaknesses. Protein quality is assessed by considering the protein digestibility of individual indispensable amino acids and their utilization (bioavailability), which is affected by food antinutrients and processing. The evidence regarding the association of protein intake per se with health outcomes is limited or suggestive. It is difficult to separate from the effect of other nutrients or ingredients in protein-rich foods. Proteins are widespread in foods, deriving from both animal and plant sources. Animal-sourced protein production puts more strain on the environment than plant-sourced proteins and contributes significantly to greenhouse gas emissions, thereby enhancing climate change. In Nordic and Baltic countries, consumption of animal-sourced proteins is relatively high. A shift toward more plant-based protein diets would be advisable for promoting a healthy and sustainable diet.

## Popular scientific summary

- Dietary proteins provide essential amino acids, nitrogen, and energy.
- Average requirements for protein intake are based on the maintenance of nitrogen balance, accounting for growth in children, pregnancy, and lactation.
- The amount and bioavailability of essential amino acids determines the protein quality of foods.
- Current intakes in Nordic and Baltic countries range from 15 to 19% of total energy intake.

Proteins are found in many food sources, including both animals and plants. Amino acids are essential for constructing proteins and various cellular structures. In nutrition, dietary protein plays two significant roles: providing a source of nitrogen (N) and amino acids and supplying energy in a non-specific manner. Proteins are especially required during active growth in pregnancy, lactation, childhood, tissue and wound healing, and tissue building in sports. National surveys conducted using varying methods in each country have revealed that protein consumption among adults in the Nordic and Baltic regions ranges from 15 to 19% of total energy intakes (E%) (1). Comprehensive systematic data from all Nordic and Baltic countries regarding children and older adults are lacking. Nevertheless, epidemiological studies with limited coverage have been conducted on children and elderly individuals (2–6). No good measure for protein status exists, and the estimation of physiological requirements is based on N-balance studies having some weaknesses (7). Protein quality is assessed considering the protein digestibility of individual indispensable amino acids and their utilization (bioavailability), which is affected by food antinutrients, natural or synthetic compounds in foods that interfere with the absorption of nutrients. The evidence regarding the association of protein intake per se with health outcomes is limited or suggestive. It is difficult to separate the effect of protein from the effect of other nutrients or ingredients in protein-rich foods.

In individuals with energy balance and moderate physical activity, the protein requirement is the lowest protein intake to maintain N-balance (8). For growing children, pregnant and lactating women, or another anabolic state, the protein requirement is taken to include the needs associated with the deposition of tissues at rates consistent with good health. In the Nordic Nutritional Recommendation (NNR), the energy content from protein in a mixed diet is calculated as 17 kJ/g.

Both animal- and plant-based foods serve as dietary protein sources. Meat, fish, milk, and eggs are major animal protein sources with specific protein content ranging from 15 to 30% (wet weight), while cereals, legumes, nuts, and seeds represent primary plant protein sources with protein content varying from 5 to 15% (dry matter) in cereals to 17 to 40% (dry matter) in legumes.

Traditionally, a nitrogen-to-protein conversion factor of 6.25 is applied to determine protein content in most foods and food ingredients. It is based on two assumptions: that the total mass of proteins contains about 16% N by weight and that all N in food is derived from protein (7). The N content of various amino acids ranges from 7.7 to 32.2%, and the N content of protein in individual foods depends on the amino acid composition. Thus, the conversion factor can vary from 5.70 for soya to 6.32 for milk. Therefore, using the same conversion factor for all protein sources can lead to significant errors in the actual protein content of most foods. Foods also contain various amounts of non-protein nitrogenous compounds, which is an additional source of error. Multiplying total N content with the default value of 6.25 to quantify protein is imperfect and can lead to a 15–20% error in the actual protein content. Protein source–specific nitrogen-to-protein conversion factors have been presented and used over the years. Still, there are no standardized methods to determine them (8), which may be why most trade and food regulatory bodies, including European Union (EU) labeling legislation, continue to use the default value of 6.25.

Amino acids from dietary proteins are classified as essential amino acids that cannot be synthesized in the human body or non-essential amino acids synthesized within the body from other amino acids (transamination) if there is an adequate N supply (Table 1).

**Table 1:** Amino acids classification

Essential amino acids	Non-essential amino acids	Conditionally essential amino acids
Isoleucine, leucine, lysine, methionine, phenylalanine, threonine, tryptophan, valine, and histidine	Alanine, arginine, asparagine, aspartic acid, cysteine, glutamine, glutamic acid, glycine, proline, serine, and tyrosine	Arginine, cysteine, glutamine, glycine, proline, and tyrosine

Histidine is considered essential, although it does not fulfill the criterion of reducing protein deposition and inducing negative nitrogen balance when removed from the diet. Conditionally essential amino acids are amino acids whose synthesis requires the availability of another amino acid either as the carbon donor or as the donor of an accessory group, for example, the sulfur group of cysteine (9). Under normal conditions, conditionally essential amino acids are synthesized in sufficient amounts. Still, during certain conditions, such as prematurity (10) and illness (11–13), the synthesis might not support all the body’s metabolic needs, which means they can become essential amino acids.

This scoping review aims to describe the totality of the evidence regarding the role of protein in health-related outcomes as a basis for setting up and updating dietary reference values (DRVs).

## Methods

This scoping review follows the protocol developed within the NNR2023 (14). The sources of evidence used in the review follow the eligibility criteria described in the paper ‘The Nordic Nutrition Recommendations 2022 – Principles and Methodologies’, published in Food & Nutrition Research (15).

For the NNR2023 update, a literature search on proteins was performed according to principles and methodology for the NNR2023, resulting in 115 systematic reviews (SRs); 38 SRs were considered relevant for three topics for SR. The literature search included the following terms: (protein[Title] AND (systematic review[Publication Type] AND (“2011”[PDAT] : “3000”[PDAT]))) AND Humans[Filter]) AND ((“diet”[MeSH Terms] OR “diet”[All Fields]) OR (“diet”[MeSH Terms] OR “diet”[All Fields] OR “dietary”[All Fields]) OR (“food”[MeSH Terms] OR “food”[All Fields]) OR (“nutritional status”[MeSH Terms] OR (“nutritional”[All Fields] AND “status”[All Fields]) OR “nutritional status”[All Fields] OR “nutrition”[All Fields] OR “nutritional sciences”[MeSH Terms] OR (“nutritional”[All Fields] AND “sciences”[All Fields]) OR “nutritional sciences”[All Fields]) OR Nutritional[All Fields]).

## Physiology and metabolism

For the body to utilize dietary proteins, they must be broken down into small peptides and amino acids. Protein digestion starts in the stomach with the denaturation of polypeptides by stomach acids and partial hydrolysis of peptide linkages by the enzyme pepsin (16). The polypeptides enter the duodenum, where they are subjected to pancreatic proteases and hydrolyzed to oligo-, tri-, and dipeptides and, to a lesser extent, to free amino acids. Peptide fragments are further hydrolyzed by brush border-bound aminopeptidases and carboxypeptidases on the luminal surface of enterocytes. The resulting free amino acids, dipeptides, and tripeptides are then absorbed through different transport pathways into an enterocyte, where cytosolic aminopeptidases hydrolyze the remaining di- and tripeptides to free amino acids. These amino acids enter the bloodstream and are incorporated into tissue protein and other N-containing compounds such as neurotransmitters, hormones, creatinine, and drug elimination ligands. This makes dietary protein requirement a requirement for amino acids and N.

Protein quality is primarily characterized by its essential amino acid content and the digestibility and availability of the resulting amino acids in the circulation. These factors influence their metabolism within different body protein pools (17). Hence, determining protein quality is based on protein digestibility ranking methods such as the protein digestibility-corrected amino acid score (PDCAAS) used earlier and the digestible indispensable amino acid score (DIAAS) currently recommended by the Food and Agriculture Organization of the United Nations (FAO) (7, 18). The significant differences between the two approaches are that DIAAS uses ileal digestibility instead of fecal digestibility and does not truncate the values artificially to 100%. DIAAS is defined as:

DIAAS % = 100 × (mg of digestible dietary indispensable amino acid in 1 g of the dietary protein/mg of the same nutritional indispensable amino acid in 1g of the reference protein).

For calculating DIAAS, it is recommended to use actual ileal digestibility values of individual amino acids, preferably measured in humans, but if not available, then values obtained in pigs or rats. As measuring ileal digestibility is highly invasive, human data still need improvement (19). Table 2 shows DIAAS values and limiting AA for various animal and plant-based protein sources (20).

**Table 2:** DIAAS % and limiting AA for animal and plant proteins (20)

Protein source	DIAAS %	Limiting AA
Corn	43	Lysine
Rice	56	Lysine
Wheat	56	Lysine
Hemp	56	Lysine
Faba bean	64	Methionine + cysteine
Oat	68	Lysine
Rapeseed	79	Lysine
Lupin	83	Methionine + cysteine
Pea	83	Methionine + cysteine
Canola	85	Lysine
Soy	103	NA
Potato	125	NA
Whey	106	NA
Egg	111	NA
Casein	137	NA
Pork	126	NA

Bioavailability comprises the proportion of the amino acid in a utilizable form and the exclusion of metabolism-interfering compounds in the food. Unprocessed plant protein sources contain antinutrients such as phytates, tannins, and protease inhibitors, which interfere with the digestion of plant proteins, making them less well-digestible and less bioavailable than animal-sourced proteins (21). Antinutrients partly explain the low DIAAS values of some plant protein sources in Table 2. The bioavailability of plant proteins varies and is generally lower than that of animal proteins. Soy and potato protein represents a good-quality plant protein with all essential amino acids, followed by legumes low in sulfur-containing amino acids and cereals low in lysine. Food matrix and food processing, for example, heating, fermentation, and extrusion, can also impact amino acid bioavailability, either increasing or decreasing it (22). It is worth emphasizing that the digestibility of individual amino acids in the same food source differs. Therefore, FAO recommends treating each amino acid as an individual nutrient when evaluating protein quality (7).

In practice, the differences in quality between proteins might be less critical in diets containing a variety of protein sources, such as in a typical Nordic mixed diet. Dietary proteins of animal origin (meat, fish, milk, and eggs) or a combination of plant proteins from, for example, legumes and cereal grains give a good distribution of essential amino acids. With the current Nordic relatively high protein intake, replacing some animal proteins with plant proteins would probably lead to lower protein intake and lower bioavailability, but still provide enough protein and essential amino acids (23). However, protein digestibility and bioavailability may become an issue in protein transition toward more plant-based diets in vulnerable groups such as older adults, as protein bioavailability is known to decrease with age (21).

Dietary protein sources contain not only proteins but also provide other critical nutrients for health. In randomized controlled trials (RCTs), replacing animal-sourced proteins with plant-based ones in a typical Nordic diet resulted in favorable changes such as increased intakes of fiber and unsaturated fatty acids and decreased intakes of saturated fatty acids and cholesterol, which were accompanied by a decrease in blood Low density lipoprotein (LDL) cholesterol (23). However, the replacement considerably decreased intakes of vitamin B_12_ and iodine (24), and markers of bone health indicated an enhanced bone turnover with a possible increased risk for fractures (25). When modeling with population data with a more moderate change of only replacing red and processed meat with legumes, increases in the average intakes of fiber, folate, K, Mg, Cu, and Fe were observed. In contrast, intakes of saturated fat, niacin, vitamin B_12_, Se, and Zn were decreased (26). Overall, distribution shifts toward a higher probability of inadequate intakes of the studied nutrients were not observed.

The efficiency of the anabolic effects of dietary amino acids on body and muscle protein depends on the quantity and bioavailability of the amino acids and on the pattern of essential amino acids, which should cover the needs of all tissues. Furthermore, the kinetics of the delivery of amino acids to muscle tissues also appears to be an essential factor in modulating the anabolic efficiency of dietary amino acids in muscle protein synthesis (11). It is assumed that the postprandial rise in circulating amino acids represents the main driver of the muscle protein synthetic response to feeding. A high rate of protein digestion, amino acid absorption, and blood amino acid delivery has been previously observed with soluble proteins such as whey protein or with different hydrolyzed proteins with a high rate of digestion and amino acid delivery (18).

Some studies suggested that a spread feeding pattern with about 30 g of dietary protein during the main meals (i.e. breakfast, lunch, and dinner) could be a more effective strategy to counteract age-related muscle atrophy and strength loss in older adults (27, 28). The results of longitudinal studies have sparked debate as they indicate that while a varied protein intake can increase muscle mass and strength, it may not necessarily improve mobility over 2 years.

**Fig. 1:**
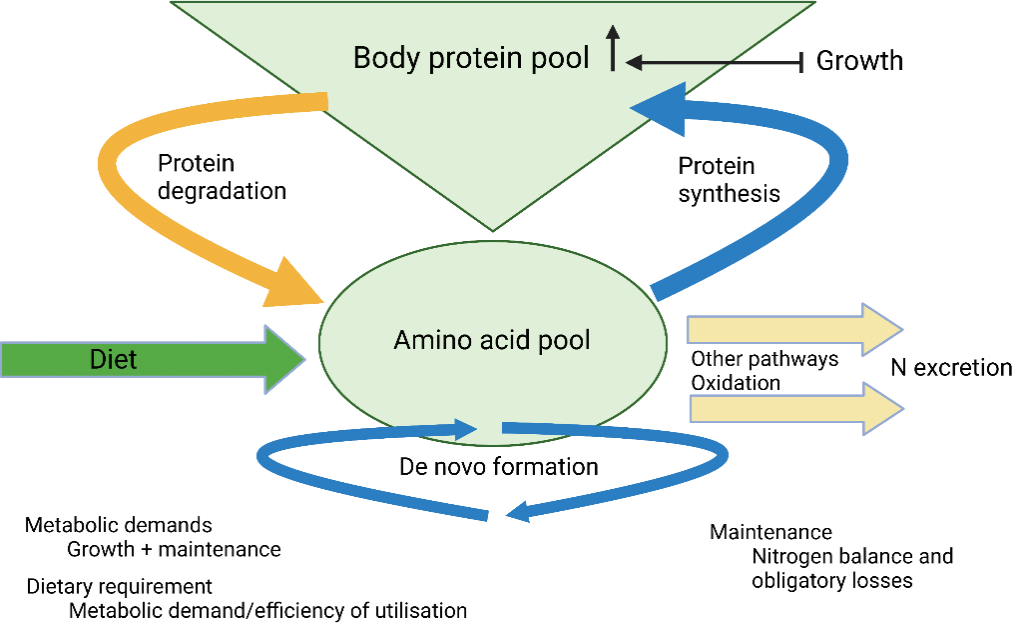
An overview of turnover of proteins and amino acids in the body, modified from Millward (30).

Body proteins, all proteins in the body, are continually broken down and synthesized. The protein turnover of about 300 g per day in adults (29) is higher than the proteins consumed from the diet (Fig. 1). This indicates an extensive reutilization of amino acids in protein metabolism. N from the amino acids leaves the body via the urine, mainly urea, uric acid, and creatinine. N is also lost in feces, sweat, and other secretions through the skin, hair, and nails. Amino acids are needed to compensate for these losses, and amino acids are also required for protein synthesis during anabolism (20), for example, during active growth in pregnancy, lactation, childhood, tissue and wound healing, and tissue building in sports. It is usually assumed that almost all dietary N is incorporated into protein. N-balance is the difference between nitrogen intake and nitrogen output. A negative N-balance, that is, losses greater than intake, is seen during fasting, starvation, and catabolic diseases like cancer, heart failure, and chronic obstructive pulmonary disease. On a long-term basis, a healthy adult should be in N equilibrium, that is, intake and losses should be equal.

### Assessment of nutrient status

There are no specific blood tests for protein deficiency or other excellent or valid measures for protein status in healthy or subclinical malnutrition. Where plasma albumin and other plasma proteins decrease in severe malnutrition, hypoalbuminemia’s etiology is multifactorial and not only related to nutrition (31). Clinical studies suggest that the underlying cause of hypoalbuminemia, rather than solely low albumin levels, is accountable for the associated morbidity and mortality (32, 33).

N-balance has been used to establish dietary protein recommendations, but its use is controversial. This methodology indirectly determines protein turnover, and no information about whole-body N, protein turnover, or various protein metabolic pathways can be obtained. It is important to consume equicaloric diets to meet nutritional needs. The World Health Organization (WHO) has examined stable isotope studies to determine the necessary intake of essential amino acids (34). Their research shows that when the oxidation of essential amino acids increases, the intake exceeds the requirements. Therefore, it is crucial to avoid low protein intake as it may lead to underestimation of our needs by inducing protein sparing (34, 35). Over the last several years, more direct methods of measuring the turnover of various body proteins have been developed, including stable isotope tracer methods. This has enabled a mechanistic approach to the effects of different dietary proteins (36). Similar logical reasoning cannot be applied to whole-body protein turnover beyond what can already be deduced from N-balance studies. Studies of muscle protein turnover have yet to add to an understanding of muscle function because no studies are available demonstrating a correlation between, for example, muscle strength or endurance and the dynamics of muscle protein turnover. Thus, in the future, it will be essential to use more advanced methodologies in strictly controlled long-term studies to establish mechanistic links between health outcomes and protein intake from various sources, as well as for different age groups and health conditions.

### Dietary intake in Nordic and Baltic countries

According to national dietary surveys, the average protein intake among adults is high in the Nordic and Baltic countries, ranging from 15 E% in females in Denmark to 18 E% in Iceland, Norway, and Finland (1) (Tables 3a, 3b). Dietary protein intake in the Nordic and Baltic countries is mainly with high-quality protein where protein contains all essential amino acids in the right proportion required by the human body, that is, a protein with high biological value. When comparing the intakes between the countries, it needs to be mentioned that while these are the most recent reliable data on the national consumption of adults, the national dietary surveys have used different methods. Therefore, the values should be compared with caution (1).

**Table 3a:** Daily mean intakes of protein among adults and children in Nordic countries (1)

	Denmark 2011 (18–75 years)		Finland 2017 (18–74 years)		Iceland 2019–2021 (37) (18–80 years)		Norway 2010 (18–70 years)		Sweden 2010 (18–80 years)	
	Men (*n* = 1,464)	Women (*n* = 1,552)	Men (*n* = 780)	Women (*n* = 875)	Men (*n* = 632)	Women (*n* = 680)	Men (*n* = 862)	Women (*n* = 925)	Men (*n* = 792)	Women (*n* = 1,005)
Protein g/day	101	76	98	73	104	76	112	81	92	72
Protein E%	16	15	18	18	19	18	18	18	17	17

	Men age 65–75 years	Women age 65–75 years	Men age 65–74 years	Women age 65–74 years	Men age 61–80 years	Women age 61–80 years	Men age 61–80 years	Women age 60–70 years	Men age 65–80 years	Women age 65–80 years

Protein g/day	95	74	76	66	96	78	102	77	84	70
Protein E%	15	15	17	17	19	18	18	18	17	17

**Table 3b:** Daily mean intakes of protein among adults and children in Baltic countries (1)

	Estonia 2014 (age 18–74 years)		Latvia 2018 (age 19–64 years)		Lithuania 2019 (age 19–75 years)	
	Men (*n* = 907)	Women (*n* = 1,806)	Men (*n* = 470)	Women (*n* = 541)	Men (*n* = 1,348)	Women (*n* = 1,562)
Protein g/day	86.5	63.2	104.4	75.1	78.7	62.8
Protein E%	16.6	16.2	17.4	17.0	15.6	16.0

	Estonia 2014 (age 70–74 years)		Latvia 2018 (age 50–64 years)		Lithuania 2019 (age 65–75 years)	
	Men	Women	Men	Women	Men and women	

Protein g/day	74.6	55.3	100.8	77.1	57.2	
Protein E%	16.7	16.5	17.8	18.1	15.1	

The average protein intake among children varies between 13 and 16 E% from about 1 year of age in most European countries, including the Nordic countries (1) (Tables 4a, 4b).

**Table 4a:** Protein intake among children in Nordic countries

Denmark			Finland			Norway		Sweden		
Age (years)	Boys	Girls	Age (years)	Boys	Girls	Age (years)	Both boys and girls	Age (years)	Boys	Girls
4–5	66^a^	58^a^	3–4	56^a^	51^a^					
	14^b^	14^b^		17^b^	17^b^					
6–9	74^a^	67^a^	5–6	61^a^	55^a^					
	14^b^	14^b^		16^b^	16^b^					
10–13	85^a^	70^a^				10–11	68^a^	12	85^a^	78^a^
	15^b^	15^b^					16^b^		18^b^	17^b^
14–17	96^a^	63^a^				14–15	75^a^	15	104^a^	79^a^
	16^b^	15^b^					16^b^		17^b^	16^b^
								18	112^a^	79^a^
									18^b^	16^b^

a g/d.

b E%.

**Table 4b:** Protein intake among children in Baltic countries

Estonia			Lithuania	
Age (years)	Boys	Girls	Age (years)	Both boys and girls
2–5	50.6^a^	47.6^a^		
	13.9^b^	14.0^b^		
6–9	66.8^a^	56.0^a^	7–10	56.3^a^
	14.6^b^	13.8^b^		15.9^b^
10–13	73.2^a^	54.0^a^	11–14	65.2^a^
	14.6^b^	13.6^b^		14.2^b^
14–17	84.5^a^	56.3^a^	15–18(19)	70.5^a^
	15.1^b^	14.5^b^		14.2^b^

a g/d.

b E%.

## Protein intake and health outcomes relevant for Nordic and Baltic countries

Severe protein deficiency results in edema, muscle weakness, and changes to the hair and skin. Protein deficiency is often linked to energy deficiency, protein-energy malnutrition, and other nutrients based on a general nutrition deficiency.

### Obesity

Many meta-analyses of short-term studies indicate favorable effects of higher versus lower protein diets on health outcomes like adiposity (38). A meta-analysis of RCTs was done in 2013 on long-term effects (≥12 months) of low- or high-protein diets on cardiovascular and metabolic risk factors (39). It showed that high-protein diets exerted neither specific beneficial nor detrimental effects on obesity.

The ‘early protein hypothesis’ established by Rolland-Cachera suggests that consuming a high amount of protein during infancy can potentially lead to an increased risk of obesity (40). The composition of macronutrients in infant formula differs from that in human milk. Protein consumption stimulates the production of insulin-like growth factor 1 (IGF-1) and insulin. Studies have shown an inverse correlation between IGF-1 levels in infancy and those in late adolescence (41). The variety of proteins ingested can have varying effects on IGF-1. In particular, the consumption of cow’s milk during early infancy has been shown to enhance linear growth and increase circulating IGF-1 levels in well-nourished populations (42). Research has shown that consuming poor-quality protein without animal protein is more likely to lead to non-communicable diseases (NCDs) later in life than consuming high amounts of total protein. Additionally, children who have a higher BMI for their age or become obese during childhood are at an increased risk of developing adult obesity and associated health problems (43).

### Cardiovascular diseases and diabetes

Cardiovascular disease (CVD) and type 2 diabetes (T2D) are significant causes of morbidity and mortality worldwide and are associated with high societal costs (3).

Based on three SRs of cohort studies by Tian et al. (44), Virtanen et al. (45), and Chen et al. (46), total and animal protein intake increased the risk of T2D, whereas plant protein decreased the risk of T2D. Again, it should be emphasized that low carbohydrate or high-protein diets cannot be used to assess the effect of protein per se.

The association between dietary protein intake from different sources of proteins and the risk of CVD in an SR of prospective studies is of interest where some studies showed no association between dietary protein intake and CVD risk from different sources of proteins (47, 48). According to a meta-analysis done in 2023, there were no observed cardiovascular effects resulting from a high-protein diet in which protein accounted for 18% or more of energy intake (49). Still, in subgroup analysis, there was a decreased risk of CVD mortality with an increasing plant protein intake. A *de novo* SR for NNR2023 by Lamberg et al. (50) concluded that evidence that substituting animal protein with plant protein reduces the risk of CVD mortality or T2D incidence is *limited – suggestive*.

Replacing animal protein with plant protein for nutrients, fibers, and sustainability should also be a public health strategy to lower the risk of CVD mortality and T2D.

### Bone health

The role of dietary protein on bone health has been controversial. Urinary calcium loss increases in high protein intakes, but at the same time, protein increases calcium absorption and bioavailability. These seemingly contradictory effects make it uncertain what the net effect of a high-protein diet (typically >20 E% and/or >1.0 g/kg body weight [BW]) is on calcium metabolism and bone health (51). Few studies have shown that protein intake above 0.8 g/kg BW prevents bone loss and hip fracture. However, the European Food Safety Authority (EFSA) (52) found that evidence is inconclusive regarding protein and bone health (53). However, there is an interaction between low calcium (<800 mg/day) intake and protein intake, where an increased risk of fractures was related to high animal protein intake. However, under high calcium intake (>800 mg), a high animal protein intake was related to a decreased risk of fractures (25). This finding is supported by an older Norwegian study of 39,787 middle-aged men and women that showed an elevated risk of hip fractures in women with high animal (non-dairy) protein intake under low calcium intake (54). The evidence for an association between vegetable protein intake and fracture risk is inconclusive (53, 55) and this finding was supported by an older study of 32,050 postmenopausal women that showed a decreased risk of hip fracture related to a high animal protein intake, but not to vegetable protein intake (56). In older adults, Pedersen and Cederholm (57) assessed the evidence as suggestive regarding a positive association between protein intake and bone mineral density based on one intervention study and three prospective cohort studies. The evidence was assessed as inconclusive regarding the relation of protein intake to bone loss and the risk of fractures. Interestingly, in the included randomized controlled study with calcium (500 g/day) and vitamin D supplementation (17.5 g/day) by Dawson-Hughes and Harris (53), the highest tertile of protein intake (20 E%, or 1.2 g/kg BW) was associated with less bone loss compared to the lowest tertile (14 E%, or 1.1 g/kg BW), but only in the intervention group. The habitual mean intake in the placebo group was 871 mg of calcium and about 7 μg vitamin D daily compared to 1,346 mg per day calcium in the intervention group. Thus, the possible effect of protein intake on bone health might depend on an input of calcium and vitamin D above this level.

### Renal function and kidney stones

High-protein diets have been associated with increased Glomerular filtration rate (GFR), serum urea, urinary calcium excretion, and serum concentrations of uric acid. Some results have indicated caution in recommending high-protein diets. However, the evidence for associations between protein intake and kidney function and kidney stones in healthy people needs to be more conclusive (35, 56, 58, 59). Also, the EFSA (52) concluded that no maximum protein intake level could be established for those with healthy kidney function due to insufficient evidence.

### Cancer

A meta-analysis from 2017 of 21 observational studies comprising 8,187 cases by Lai et al. (60) found no association between protein intake or protein source (animal vs. plant) and colorectal cancer risk. The authors reported a significant between-study heterogeneity, which may have affected the results. Liao et al. (61) utilized a large prospective US cohort comprising nearly a half-million adults with a median follow-up of 15.5 years to evaluate the effect of substituting plant protein for animal protein on the risk of colorectal cancer. The substitution was associated with up to an 11% decreased risk of colorectal cancer, primarily due to substituting plant proteins for red meat, not white meat, dairy, or egg protein. Further analyses revealed that the risk reduction was mostly limited to replacing protein from bread, cereal, and pasta for red meat protein and was statistically significant only for the distal colon and rectum. These results align with other studies and the latest World Cancer Research Fund’s food-based evaluation, concluding that solid scientific evidence exists, suggesting that high intakes of red and processed meat increase, and high intakes of dairy products and whole grains decrease, the risk of colorectal cancer (62).

A meta-analysis of 11 studies with 2,537 cases and 11,432 participants by Kong et al. (2020) (63) found no association between overall dietary protein intake and esophageal cancer risk. Still, a sub-analysis revealed an increased risk for esophageal squamous cell carcinoma.

No association was found between prostate cancer and dietary protein intake, or animal or plant protein intake in a meta-analysis of 12 studies comprising 13,483 prostate cancer cases and 286,245 participants (64). The authors claim no publication bias or between-study heterogeneity.

A meta-analysis of 46 prospective studies by Wu et al. (65) examined the association between dietary protein sources and breast cancer risk. They found that a higher intake of soy food and skim milk could decrease the risk and that a higher intake of processed meat may increase the risk of breast cancer.

Most studies on the relationship between protein intake and cancer are food-based and, therefore, cannot isolate the effect of the protein intake per se from other nutrients or ingredients in foods. Furthermore, while plant-based foods are commonly associated with a somewhat lower risk of cancers, different animal-based foods may have opposite effects on cancers, as demonstrated by red and processed meat and dairy products in colorectal cancer (62).

### Mortality

Several meta-analyses found that a high animal protein intake was positively associated with cardiovascular mortality. A high plant protein intake was inversely associated with all-cause and cardiovascular mortality, especially among individuals with at least one lifestyle risk factor. The substitution of plant protein for animal protein, especially that from processed red meat, was associated with lower mortality, suggesting the importance of protein sources (48, 66–70). An SR of cohort studies from 2020 on the connection between protein sources and mortality yielded inconsistent findings (68). Nevertheless, it has been confirmed that older men (mean age 74 years) who do not consume enough protein (<0.8 g/kg/day), regardless of its source, have a slightly higher risk of all-cause and cause-specific mortality (71). Based on current literature, replacing animal protein with plant protein may have health benefits (72).

### Requirement and recommended intakes

The metabolism of proteins in humans is closely related to energy intake (73). The WHO/FAO/UNU (34) define the protein requirement of an individual as ‘the lowest level of dietary protein intake that will balance the losses of nitrogen from the body, and thus maintain the body protein mass, in persons at energy balance with modest levels of physical activity, plus, in children or pregnant or lactating women, the needs associated with the deposition of tissues or the secretion of milk at rates consistent with good health’. Despite limitations in the method that are mainly related to the accuracy of the measurements and interpretation of the results, N-balance remains the method of choice for determining the protein requirement in adults in the absence of validated or accepted alternatives and the lack of a reliable biological marker of protein status.

### Recommended intake

#### Adults

The EFSA (52) states that 0.83 g of good-quality protein/kg BW per day based on an estimated average requirement (EAR) of 0.66/kg BW per day covers daily protein needs. The 2002 US recommendations from the Institute of Medicine (now the National Academies of Sciences, Engineering, and Medicine, NASEM) (74) for protein were also based on the meta-analysis of N-balance studies by Rand et al. (75) and cite an EAR of 0.66 g/kg BW per day and a recommended daily allowance (RDA) of 0.8 g good-quality protein/kg BW per day. These recommendations are for healthy adults based on a coefficient of variation of 12% and with no significant differences according to adult age or sex.

An SR was conducted to update the NNR2012 (57) to evaluate the potential health impacts of different protein intake levels. The evidence regarding protein intake was largely inconclusive for most outcomes, including all-cause mortality, cancer mortality and diseases, CVD, bone health, BW control, body composition, and renal function. However, the review identified a potential relationship between plant-based proteins and lower cardiovascular mortality and blood pressure rates. Thus, despite some studies finding a decreased risk of outcomes associated with vegetable protein intake (76), the authors concluded there was insufficient evidence to recommend an increased intake of plant-based sources.

Based on Nordic and Baltic dietary habits and available evidence, the current intake falls within the recommended range of 10–20% of daily energy intake (1). This protein intake adequately meets the requirements for essential amino acids. With decreasing energy intake below 8 MJ (e.g. due to decreased physical activity or during intentional weight loss), the protein E% should increase accordingly.

### Infants and children

Good nutrition is vital for healthy growth and development during the first 2 years of life. Starting good nutrition practices early can help children develop healthy dietary patterns. Regarding the later risk of NCDs such as CVD, the quantity and quality of protein intake in infancy and childhood are of interest.

Breastfeeding is associated with a reduced later obesity risk relative to feeding conventional infant formula. Breastfeeding induces less weight gain during the first 2 years of life, which predicts less obesity up to adulthood (77). During the first 6 months, infants are breastfed or receive infant formula. The protein content of breast milk is considered adequate for term infants, and the protein content of infant formula is regulated by the EU legislation. According to the current regulation/directive (REGULATION [EU] No 609/2013 and COMMISSION DIRECTIVE 2013/46/EU), the protein content of infant formula should be between 0.45 and 0.7 g/100 kJ, and the protein content of follow-on formula should be between 0.45 and 0.8 g/100 kJ.

Concerning BW, the WHO/FAO/UNU (78) gives reference values of 0.9 g/kg BW per day from 3 to 18 years of age for boys and from 3 to 15 years of age for girls. This value decreases slightly for girls to 0.8 g/kg BW per day between 15 and 18 years of age. The protein energy percentage necessary to cover the adequate protein intake can be calculated by combining these reference values with the reference values for energy intake for age and sex. The average requirement calculated as E% is about 5.3 E% at 6 months, followed by a decline to 4.3 E% at 2 years. After that, there is a gradual increase. The recommended safe level has decreased for all ages, especially for the first 2 years (Table 5).

**Table 5:** A safe level of protein intake (average requirement + 1.96 SD) in weaned infants and children (52)

Age	Protein g/kg BW	E%	g/100 kJ
6–11 months	1.1	7–15	0.4–0.9
12–23 months	1.0	10–15	0.6–0.9
2–17 years	0.9	10–20	

Expressed as E%, the protein intake increases considerably during the first 1–2 years of life when the infant gradually changes from breast milk with about 5 E% to the family diet that typically provides around 15 E% from protein. The average protein intake among children varies between 13 and 16 E% from about 1 year of age in most European countries, including the Nordic and Baltic countries (52). In the Nordic setting, quantity is more important than quality because the protein sources are usually of animal origin.

The appropriate upper limit for protein intake in infancy and childhood has not yet been firmly established, and there is interest in understanding the short- and long-term effects of different levels of protein intake during this period. Research has focused on growth, serum lipids, glucose and insulin, blood pressure, BW, body composition, and bone mineral density. While some studies suggest that higher protein intake during infancy and early childhood may increase the risk of obesity later in life (79, 80), results have been inconclusive for other long-term health parameters (81). Available data show that a protein intake between 15 and 20 E% during the first 2 years of life may increase the risk of being overweight later in life (43).

There is suggestive evidence that animal protein intake, especially from dairy products, has a stronger association with growth, particularly with weight gain, than plant protein. The evidence also suggests that a higher animal protein intake in childhood was associated with earlier onset of puberty (82, 83).

### Pregnant and lactating women

During pregnancy, the average protein requirement is increased to provide additional protein for deposition in maternal (blood, uterus, and breasts), fetal, and placental tissues. Extra protein is also needed to maintain the increased mass of the pregnant body. According to the EFSA report (35), pregnant women should consume 1, 9, and 28 g of protein per day during their first, second, and third trimesters, respectively, in addition to the recommended protein intake for non-pregnant women. On the other hand, the German recommendations (84) for protein intake during pregnancy are comparatively lower than those of the EFSA, as they suggest an additional 10 g per day during the second and third trimesters.

However, increased protein intake during pregnancy – due to increased energy intake – should consist of regular food rather than high-protein supplements.

The average protein requirement is also increased during lactation when the breast milk produced by a woman provides all the protein her infant needs. The EFSA (35) recommends as a safe level of additional protein for lactating women an additional 19 g protein per day during the first 6 months of lactation (exclusive breastfeeding) and 13 g protein per day after 6 months (partial breastfeeding) above the recommended intake for non-lactating women. Therefore, a lactating and pregnant woman can, in most cases, cover the protein requirements with a regular diet if energy requirements are covered.

### Older adults

As people grow older, their vulnerability to chronic illnesses increases. Nevertheless, it is essential to acknowledge that the health and functional capabilities of individuals aged 65 years and above vary and are not solely dependent on their age. Such conditions might lead to periodic temporary loss of body protein through chronic inflammations, catabolic exacerbations of the disease, brief periods of bed rest, and loss of appetite. The losses must be replaced from the diet, thus representing an added need for dietary protein (85). In addition, older individuals exhibit a gradual loss of muscle mass and strength with age. This is estimated to be a daily loss of 0.5 mg N per kg BW (86) that occurs naturally and is not simply due to decreased physical activity (87).

Frailty (88) and sarcopenia (89) indicate an increased risk of developing adverse health outcomes such as the onset of disability, morbidity, institutionalization, or mortality (90). Physical function, or physical performance, is the clinically relevant outcome of muscle mass. An SR and meta-analysis of observational studies by Coelho-Júnior et al. (91) to investigate the association of relative protein intake and physical function in older adults showed that high protein intake (≥1.2 g/kg/day) and protein intake ≥1.0 g/kg per day among older adults resulted in better lower limb physical functioning and walking speed performance, respectively, in comparison to individuals who present relatively low protein (<0.80 g/kg/day) intake. The authors concluded that these findings provided additional evidence for increasing dietary protein recommendations for community-dwelling older adults. Other studies on protein supplementation and resistance exercise in healthy older adults or with sarcopenia or frailty support these findings (91, 92). Pooled data (93) from European and North American community-dwelling older adults showed that higher daily protein intake might reduce physical function decline not only in older adults with protein intake below the current recommended dietary allowance of 0.8 g/kg BW per day, but also in those with a protein intake that is already considered sufficient, that is, above and beyond 0.8 g/kg BW per day. This dose-dependent association was observed for all levels of physical activity, suggesting that the results on the association between protein intake and physical function do not change by physical activity level. Other studies have shown an association between protein intake and lean muscle mass. However, other studies in healthy populations with protein supplements and physical training have not shown muscle augmentation (94, 95).

Results from prospective cohort studies suggested that a safe intake of up to 1.2–1.5 g protein/kg BW per day or approximately 15–20 E% represents an optimal intake level. Concerning the age-related decrease in energy intake, a diet with protein content in the range of 10–14 E% might not sufficiently cover the need for protein in absolute amounts. It is important to note that the older adult population is diverse, and individuals aged 65–75 years are generally considered healthy and able to maintain their energy levels. The recommendation from NNR2012 (96) for food planning was 18 E%, corresponding to about 1.2 g protein/kg BW per day. This recommendation aligns with several expert groups (94, 97) which have suggested a daily amount of 1.0–1.2 g/kg BW per day for healthy older adults. The ESPEN guidelines (98) recommend that protein intake be at least 1 g/kg BW per day and up to 2 g/kg BW per day for illness, injury, or malnutrition. However, an SR by the health council of the Netherlands concluded that increased protein intake has a possibly beneficial effect on lean body mass and, when combined with physical exercise, muscle strength; likely no effect on muscle strength when not combined with physical exercise, or on physical performance and bone health; an ambiguous effect on serum lipids; and that too few RCTs were available to allow for conclusions on the other outcomes. This SR provides insufficient evidence that increasing protein in older adults with a protein intake ≥0.8 g/kg BW per day elicits health benefits (99).

### Upper intake levels

The WHO (34) and NASEM (74) recommend that individuals without chronic kidney disease can safely consume higher-protein diets. The upper limit for protein intake in adults in NNR2012 was 20 E%. It was suggested to consume the following upper limits of protein, assuming adequate intake of other essential nutrients: 10 E% for 0–6 months, 15 E% for 6–11 months, 17 E% for 12–23 months, and 20 E% for 2 years and above.

Based on the risk of mortality and morbidity, the SR by Pedersen et al. (57) also assessed the evidence for a potential adverse effect of a high protein intake. There was no indication of adverse effects of protein intake concerning bone health provided a sufficient calcium intake, and an included meta-analysis did not find support for the acid hypothesis that a diet high in animal protein results in an increased systemic acid load and causes osteoporosis (100). One study with elderly subjects (101) found that animal protein intake was related to an increased risk of hypertension among persons ≥70 years of age. The lowest tertile of total protein intake was 14 E%, and the highest was 19 E%. An increase in GFR is a physiological adaptation to increased protein intake (102). Walrand et al. (103) found that a high protein intake did not increase GFR in the elderly participants in their study from a baseline GFR lower than that of the young participants. This was probably due to reduced kidney function in older adults. Individuals with mild-to-moderate chronic kidney disease also do not show the usual protein-induced increase in GFR (104). Caution is also required due to the observation of a decline in GFR among women with mild kidney insufficiency (105) and because older adults might have undiagnosed compromised kidney function due to severely reduced GFR. Regarding microalbuminuria, one experimental study found an increase in urinary albumin after seven days of a high protein intake of 2.4 g/kg BW per day. However, a similar increase in protein intake in another short-term experimental study of healthy young men did not find an increase in 24-h urinary albumin excretion (35). Further studies are needed to settle whether this discrepancy is due to the different durations of the studies or to various methods of analysis of albumin in the urine. Friedman (106) cites an earlier 3-week study showing a reduction in proteinuria with reduced protein intake (75–43 g per day). Caution is required until this matter is settled.

### Protein and physical exercise

According to a position paper from the Academy of Nutrition and Dietetics, Dietitians of Canada, and the American College of Sports Medicine from 2016 (107), co-ingestion of protein with carbohydrates during recovery results in improved net protein balance post-exercise. Ingesting approximately 20–30 g total protein, or approximately 10 g of non-indispensable amino acids during exercise or the recovery period (post-exercise), seems to increase whole-body and muscle protein synthesis and improve N-balance. Current data suggest that dietary protein intake necessary to support metabolic adaptation, repair, remodeling, and protein turnover generally ranges from 1.2 up to 2.0 g/kg per day in cases of energy restriction or elevated protein intakes as high because of injury (102). Daily protein intake goals for the general population can be met with a meal plan providing a regular distribution of moderate amounts of high-quality protein across the day and following strenuous training sessions.

To match energy expenditure, adequate energy consumption, particularly from carbohydrates, is vital so amino acids are spared for protein synthesis and are not oxidized (108). According to Morton et al. (109), after reviewing 49 studies with 1,863 participants, it was found that dietary protein supplementation can greatly improve muscle strength and size changes during prolonged resistance exercise in healthy adults. However, consuming more than ~1.6 g/kg per day of protein through supplementation does not lead to additional gains in fat-free mass induced by resistance training.

A meta-analysis by Messina et al. (110) with 9 studies and 266 participants showed no difference between the effects of supplementing with soy protein and animal protein on gains in muscle mass and strength in response to resistance exercise, which is interesting in the transition toward plant-based protein sources, especially among young people.

Although animal protein is usually considered a more potent stimulator of muscle protein synthesis than plant protein, the effect of protein sources on lean mass and muscle strength must be systematically reviewed, considering more sustainable protein sources. One meta-analysis of 16 studies where total protein intakes were generally above the recommended dietary allowance at baseline and end of intervention showed that protein source did not affect changes in absolute lean mass or muscle strength. However, the authors concluded that animal protein tends to be more beneficial for lean mass than plant protein, especially in younger adults (<50 years old) (111).
